# Supplementary material for: Onset of criticality in hyper-auxetic polymer networks
Source: Nat Commun. 2022 Jan 26;13:527. doi: 10.1038/s41467-022-28026-z (PMC8791937; doi:10.1038/s41467-022-28026-z)
Supplement: Supplementary file 1 — Supplementary Information [file 41467_2022_28026_MOESM1_ESM.pdf]

Supplementary Information for: Onset of criticality in hyper-auxetic polymer networks

Andrea Ninarello,* José Ruiz-Franco,* and Emanuela Zaccarelli†
CNR Institute of Complex Systems, Uos Sapienza, Piazzale Aldo Moro 2, 00185, Roma, Italy and
Department of Physics, Sapienza University of Rome, Piazzale Aldo Moro 2, 00185 Roma, Italy

SI. ELASTIC MODULI AT ULTRA-LOW CROSSLINKING: COMPARISON WITH LINEAR ELASTICITY THEORY

In Fig. S1 we report the bulk K and Young Y moduli as a function of pressure P for a diamond topology, with different crosslinker concentrations c. Both moduli at low c display a minimum, which becomes more pronounced upon further lowering c, at a characteristic (negative) value of P. Such values appear to be close but not exactly the same, within the current numerical resolution, as P_{min}, where the Poisson's ratio has a minimum, as discussed in the main text.

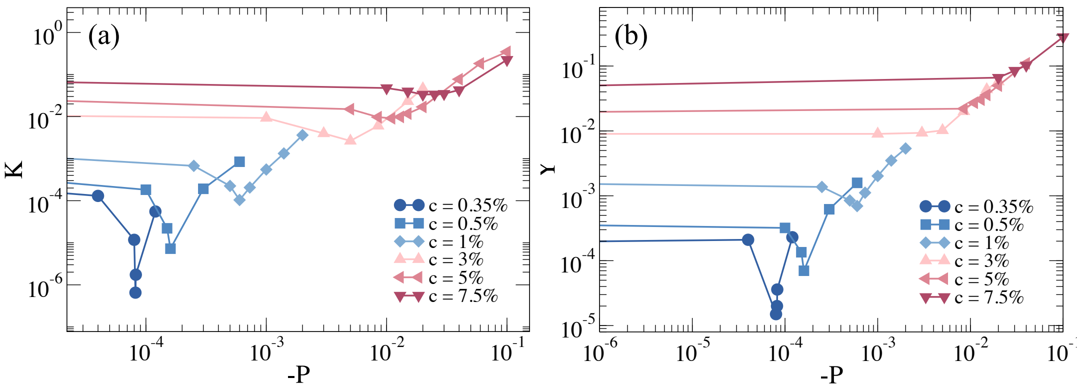

Figure S1: (a) Bulk modulus K (a) and (b) Young modulus as a function of pressure for diamond networks with different crosslinker concentrations. Both moduli are given units of $k_B T/\sigma^3$ (see Methods in the main text).

* These authors contributed equally
† Corresponding author:emanuela.zaccarelli@cnr.it

SII. DEPENDENCE ON NETWORK TOPOLOGY FOR DISORDERED REALIZATIONS

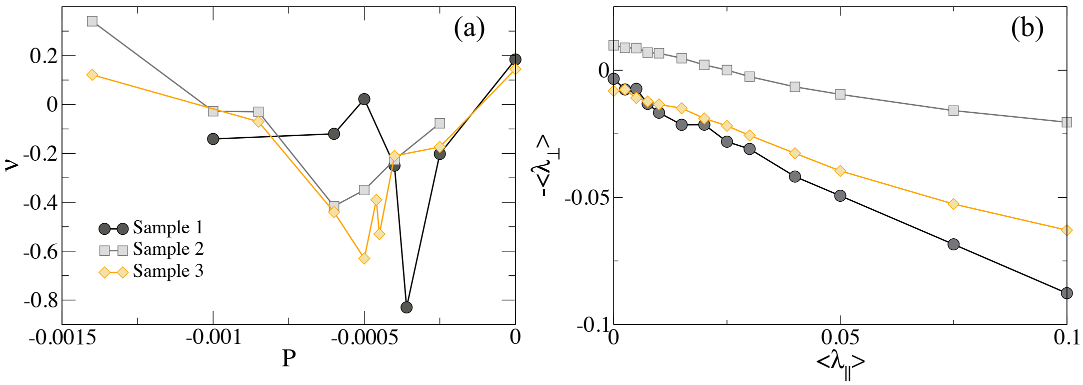

Figure S2: Stress-strain simulation results for the Dis - 1% network for three different topologies: (a) Poisson's ratio versus negative pressure; (b) negative transverse strain as a function of parallel strain for each topology at their respective $P_{min} = -3.6 \times 10^{-4}, -5 \times 10^{-4}, -6 \times 10^{-4} k_B T/\sigma^3$ for topology $1, 2$ and 3, respectively.

In Fig. S2 we report results for the Poisson's ratio for different realizations of the Dis - 1% network, to show that we find consistent behaviour independent of the specific topology. In particular, Fig. S2(a) shows ν as a function of pressure for the three studied realizations, displaying a minimum in all cases, although taking place at different values of P_{min}. The transverse versus longitudinal strain for each P_{min} shows a similar behaviour for the different topologies, as reported in Fig. S2(b).

SIII. ELASTIC PROPERTIES FOR COMPRESSED AND EXPANDED (COEXISTING) STATES AT THE HYPER-AUXETIC POINT

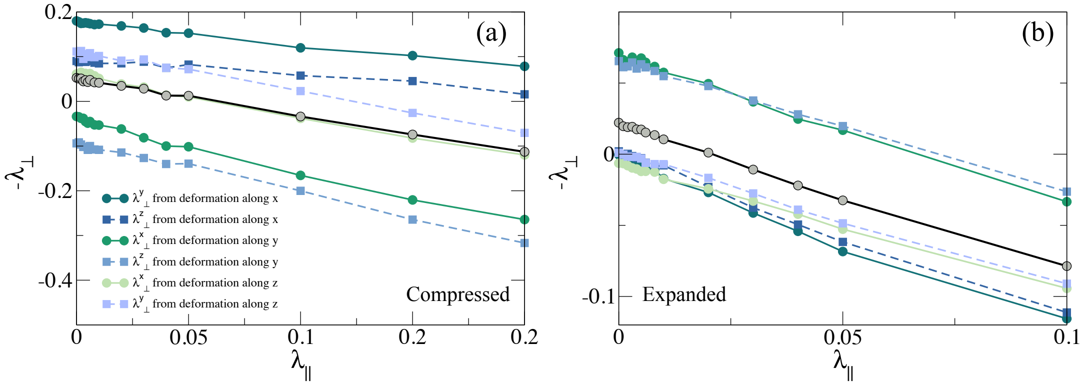

Figure S3: Results for the Diam - 0.35% network at $P_{min} = 8.19 \times 10^{-5} k_B T/\sigma^3$: negative transverse strain obtained for the three axial directions as a function of parallel strain $\lambda_{\parallel}$ both for the compressed (a) and the expanded (b) state.

Here we report results for the Poisson's ratio of the two coexisting compressed and elastic states for the Diam - 0.35% network at $P_{min} = 8.19 \times 10^{-5} k_B T/\sigma^3$, as discussed in Fig. 3 of the main text. The calculated $\lambda_{\perp}$ vs $\lambda_{\parallel}$ for the compressed and expanded states are reported in Figs. S3(a,b), respectively, for all examined deformation directions. From these data, it is evident that the Poisson's ratio does not change much between the two states, being $\nu \simeq -1$ for the expanded state and -0.84 for the compressed one. These data suggest that a hyper-auxetic behaviour is found for both states, although the bulk and the Young moduli are found to be significantly different from each other, as discussed in the main text.

SIV. CRITICAL-LIKE BEHAVIOUR: ENTROPY AND ENERGY

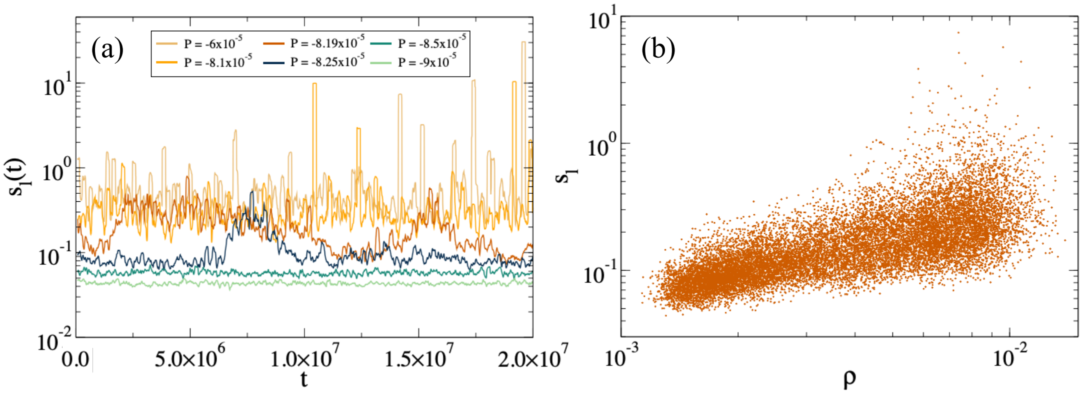

Figure S4: (a) Single chain entropy s_l as a function of time for the Diam - 0.35% at different negative pressures; (b) scatter plot of the same quantity with respect to density at $P_{min} = -8.19 \times 10^{-5} k_B T/\sigma^3$.

As discussed in the main text, entropy plays an important role in the transition. In polymeric systems, the presence of crosslinkers and entanglements induces a constraint on the configurations that can be explored by the polymer chains. Typically, the chain end-to-end distance r can be used to describe chains fluctuations, whose probability distribution has theoretically been approximated by a Gaussian distribution [1, 2]. In this way, the entropy of the network is computed as the sum of the contributions from every chain. While this approach was shown to be useful in dense systems, in dilute regimes it fails due to the presence of short chains whose r does not behave in a Gaussian way. Furthermore, by stretching the network, deviations from the gaussian behaviour will be significant [3]. To avoid these issues, here we calculate single chain entropy assuming that the end-to-end distance follows the Langevin approximation [2], which for a single chain is given by Eq. 5 of the main text. This approach has been shown to work relatively well in the case of phantom networks up to end-to-end distances approaching the contour length nb [3]. Thus, we define the average single chain entropy s_l as the average entropy of all the chains within the network which is reported in Fig. S4(a) for the Diam - 0.35% network at different negative pressures. Interestingly, we observe critical-like jumps between a high entropy regime corresponding to high density and low entropy states corresponding to low density, which happen simultaneously with those occurring for density (see Fig. 4(a) in the main text). This is confirmed by the scatter plot of entropy and density at P_{min} in Fig. S4(b).

When we monitor the total potential energy e_t as a function of time, as reported in Fig. S5(a), again for the Diam - 0.35% network at the same value of P, we find that critical fluctuations are completely absent. We thus focus on the non-bonded potential energy e_{nb}, shown in Fig. S5(b), which instead shows critical-like fluctuations. This correspondence is confirmed by the scatter plots, reported in Figs. S5(c) and (d), of total energy and non-bonded energy with density, respectively. Clearly, while the former appears to be not correlated with ρ, the second is. This analysis allows us to focus on e_{nb} as mixing variable in the order parameter M that controls the criticality of the transition, as also discussed in the main text.

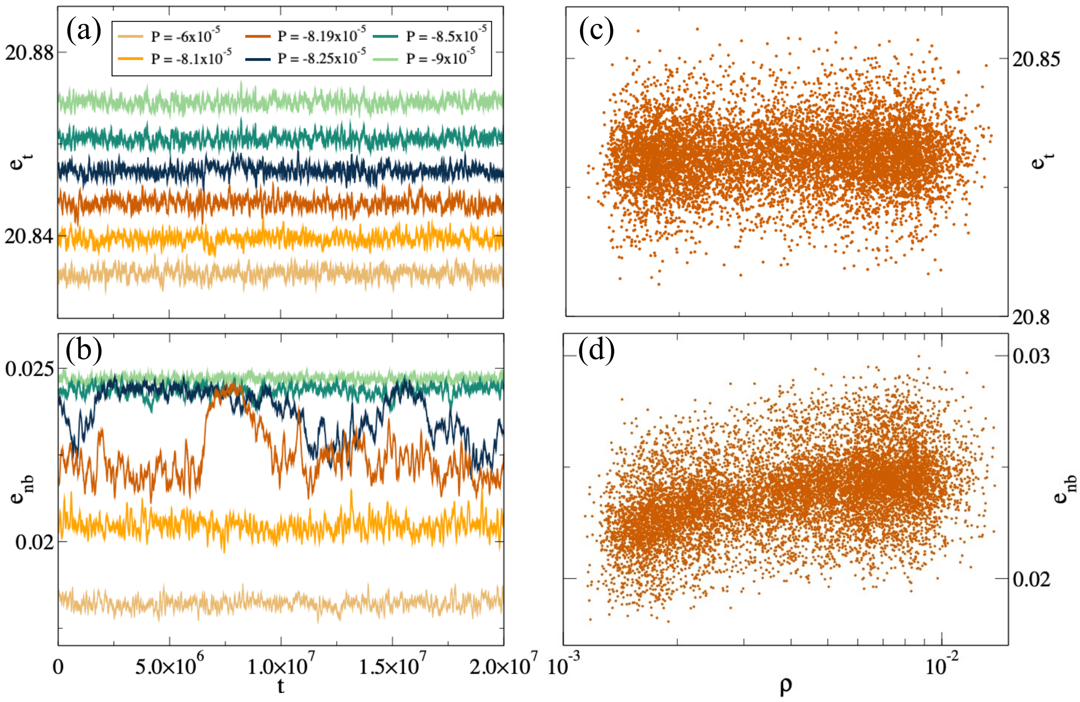

Figure S5: Energy as a function of time for the Diam - 0.35% network at different negative pressures: (a) total potential energy e_t and (b) non-bonded particle energy e_{nb}. Data for e_t are vertically shifted by 0.05 with respect to each other to improve visualization; (c, d) scatter plots of the same quantities with respect to density ρ at $P_{min} = 8.19 \times 10^{-5} k_B T/\sigma^3$.

SV. SCALING PROPERTIES CLOSE TO THE TRANSITION

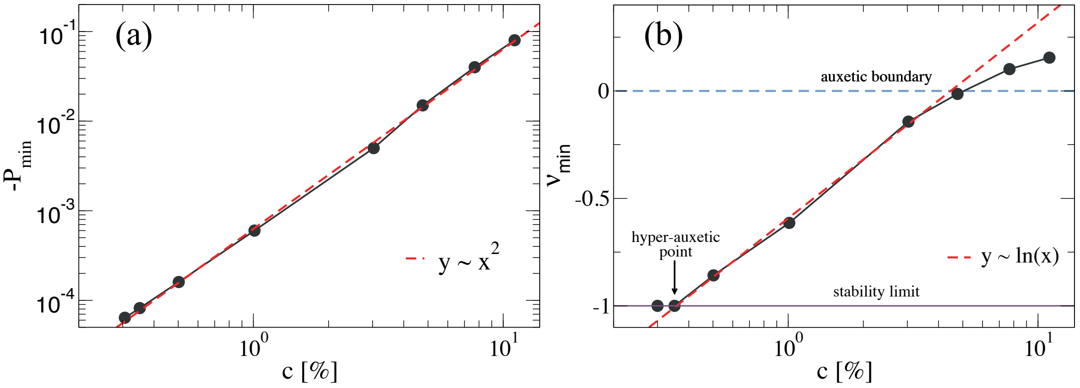

Figure S6: (a) Minimum pressure P_{min} and (b) minimum Poisson's ratio ν_{min} for diamond networks as a function of crosslinker concentration. Fits to the data are shown as dashed lines: in (a) a power law fit is used, while in (b) we employ a logarithmic function. The horizontal lines in (b) indicates $\nu = 0$ (dashed), below which auxetic behavior starts, and $\nu = -1$ (solid), that sets the limit of stability of the network. Note that for $c < c^*$ deviations from the logarithmic fit are observed, because the minimum Poisson's ratio always remains equal to -1. We do not include this last point in the fit.

In Fig S6 we report the behavior of P_{min} and ν_{min} as a function of crosslinker concentration for diamond networks. It is interesting that P_{min} closely follows a quadratic power-law behavior for $c \lesssim 1\%$, suggesting a critical behavior that would terminate at $c = 0$. Instead, ν_{min} follows a logarithmic behavior in c in the auxetic region, which ends at c^*, below which the minimum Poisson's ratio saturates at -1, the minimum value for a mechanically stable solid. For $c < c^*$ we indeed still detected $\nu_{min} = -1$, followed by a first-order transition between the two states upon further

decreasing pressure.

[1] Mark, J. E. *et al.* *Physical properties of polymers handbook*, vol. 1076 (Springer, 2007).
[2] Rubinstein, M. & Colby, R. H. *Polymer physics*, vol. 23 (Oxford university press New York, 2003).
[3] Sorichetti, V. *et al.* Effect of chain polydispersity on the elasticity of disordered polymer networks. *Macromolecules* **54**, 3769–3779 (2021).
